# Elevating Married Adolescents' Voices for Responsive Reproductive Healthcare in Syria

**DOI:** 10.3389/frph.2022.780952

**Published:** 2022-01-28

**Authors:** Pari Chowdhary, Anushka Kalyanpur, Feven Tassaw Mekuria, Ihlas Altinci

**Affiliations:** ^1^Health Equity and Rights Team, CARE USA, Atlanta, GA, United States; ^2^Sexual and Reproductive Health Team, CARE Turkey, Gaziantep, Turkey

**Keywords:** married adolescent girls, humanitarian, sexual and reproductive health, co-implementation, child marriage, first-time mothers, pregnant adolescents

## Abstract

Increases in early marriage and pregnancy resulting from Syria's humanitarian crisis highlight a critical gap in adolescents' access to life-saving sexual and reproductive health information and services, and a larger need for adolescent-specific interventions grounded in gender transformative approaches. Seeking to address this, CARE, UNFPA and Syria Relief and Development adapted global evidence-based approaches to humanitarian contexts to create the Adolescent Mothers Against all Odds (AMAL) Initiative for pregnant girls and first-time mothers aged 10 to 18 years. Designed to improve the lives of young girls through responsive health systems and enabling environments, AMAL includes three components: a Young Mothers Club for first-time mothers and pregnant girls, participatory dialogues with health providers, and reflective dialogues with girls' marital family and community members. The AMAL Initiative intends to ensure responsiveness to the unique vulnerabilities of adolescent sub-groups by co-implementing with them. Select girls undergo additional leadership training and serve as adolescent representatives on community advisory groups sharing feedback for program improvement. One hundred-four first-time mothers and pregnant girls, 219 community members, and 120 health providers participated in AMAL in northwest Syria. In a mixed methods evaluation, facilitators administered monitoring tools to identify program improvements, pre-post surveys to assess outcomes, and end-line discussions to gather perceptions of impact. Girls reported a 47% overall increase in self-esteem, confidence, health-seeking capacity, and communication ability. Community support for girls' use of family planning increased by 27% and girls' equal access to services by 35%. Findings across all participant groups demonstrate decreased expectations of early marriage and increased acceptance of family planning post-marriage. Areas that participants cited for potential improvement included programming for girls/women above the age of 18 years, and additional training for health providers on long-acting contraceptive methods. These results show that participatory adolescent-centered sexual and reproductive health programming is not only feasible in crisis settings but can improve the self-efficacy of vulnerable adolescents to overcome barriers to accessing healthcare and improving well-being. The AMAL Initiative is now being scaled up through local partners in Syria and piloted in northern Nigeria.

## Introduction

Working in crisis settings requires an understanding of the lived experiences of people, the power dynamics, and micro-politics that inform humanitarian response approaches. Of UNICEF's nine Core Humanitarian Standards, three refer specifically to ensuring accountability toward affected peoples (1). In practice this could include centralizing the voices of affected peoples by engaging communities in needs and performance assessments and decision-making. While there is consensus across global humanitarian and development actors of the need for unique programmatic focus on adolescents, there is a dearth of interventions that effectively consider and elevate adolescent voices and lived experiences (2). The Global Strategy for Women's, Children's, and Adolescent's Health (2016–2030), a roadmap for ending preventable deaths and expanding enabling environments, is being widely implemented for adolescent populations in low- and middle-income countries. In its monitoring framework, the strategy promotes accountability toward local populations to support adolescents reaching their potential (3). Given adolescents' increased vulnerability during crises, their inclusion in the design, implementation and evaluation stages of humanitarian programming is especially important. Yet, a UNFPA analysis of organizations implementing programs under this strategy determined that adolescents were rarely included in program planning processes and in the Middle East in particular, social norms of exclusion of young girls prevailed (3). To be effective and equitable toward global populations, humanitarian organizations must adhere to core standards, and carry out their efforts in a manner that is inclusive of the communities they are seeking to serve.

After a decade of conflict, Syria's humanitarian crisis remains one of the world's largest. Despite remarkable resilience, more than half the Syrian population is experiencing displacement and poverty resulting from political instability, violence, physical devastation, and environmental disasters. Within Syria, 6.7 million people are internally displaced, of which 4.4 million are women and children (4). Eighty percent of Syrian refugees are living below the poverty line, and 2.4 million children are out of school (5). Women and girls are disproportionately experiencing the burden of the conflict with high risk of sexual violence and limited access to sexual and reproductive health services (6). In northwest Syria, the situation is particularly acute. Flooding, outbreaks of violence, and destruction of infrastructure since 2019 have severely fragmented the lives of those living in the region (7). The 2020 COVID-19 pandemic further worsened the situation, restricting access to already limited relief services (8). As is common in humanitarian crises, adolescents are especially affected and vulnerable to risk. Economic hardship brought on by displacement is giving rise to negative coping mechanisms for individuals and families. School-aged children are instead being sent to work in dangerous settings, and early and forced marriage for young girls is increasing (7). While there is much evidence in the literature of the harm adolescents can experience within marriage, many Syrian communities view marriage as a mechanism to protect a girl's honor and therefore increasingly practice early marriage during times of uncertainty and violence (9, 10). A survey assessing rates of marriage among Syrian women before the age of 18 found a 34% increase in child marriage after the onset of the crisis, and determined displacement, instability, and poverty as the major driving forces behind this trend (11). Higher prevalence of early marriage renders adolescent girls additionally vulnerable increasing their risk of adolescent pregnancy, complications of pregnancy, malnutrition, and maternal mortality. This burden of disease is compounded by the disruption and destruction of health systems and infrastructure. Globally, lack of sexual and reproductive healthcare services is the leading cause of death and disease among refugee women (12). Health systems inside Syria have been debilitated through years of conflict with only 52% of primary health centers still operating and 30% of healthcare staff remaining (13, 14). A recent analysis of care provision in conflict settings found that in Syria, even basic sexual and reproductive care and services are unavailable to most of the population (15). In 2021, 3.6 million Syrian women and adolescent girls, half a million of whom are pregnant, are in need of quality sexual and reproductive services highlighting a critical gap in adolescents' access to life-saving care during this crisis (16).

Seeking to address this service gap and actualize principles of community participation in humanitarian settings, CARE, UNFPA and Syria Relief & Development created the Adolescent Mothers against All Odds (AMAL) Initiative. Designed to meet the immediate needs of pregnant adolescents and first-time mothers in crisis-affected settings, the AMAL Initiative targets community consciousness and engagement around gender, power, and social norms to support enabling structures and environments. It has three main components: Young Mothers' Clubs, Community Dialogues, and Provider Dialogues. The Young Mothers' Club is a peer-based discussion group made up of pregnant adolescents and first-time mothers centered around improving sexual and reproductive health knowledge and strengthening life skills. Community dialogue groups garner support for project activities to create enabling environments for adolescent girls, and work to influence programmatic elements to make them more adolescent-responsive. Members comprise influential individuals such as religious leaders, teachers, and community health workers as well as mothers, mothers-in-law, and husbands of adolescent girls. Provider dialogue groups engage health services providers in participatory exercises focused on rights-based approaches to family planning counseling, communication skills and ensuring adolescent-friendly health services to improve attitudes and reduce biases toward adolescent sexual and reproductive health service provision. Recognizing the capacity of adolescents to influence change for themselves, the AMAL Initiative also includes Adolescent Advisory Committees (AACs). Young Mothers' Club graduates who demonstrate interest in supporting other adolescents participate in AACs undergoing a series of leadership sessions to further facilitate their self-efficacy. AAC members play a key role in strengthening the responsiveness of the program to the needs of adolescents by (1) identifying hard-to-reach and marginalized adolescents in their communities to refer them to AMAL programming, health facilities, and other support systems, (2) sharing recommendations on creating adolescent-responsive environments with community stakeholders, and (3) providing program feedback in monthly meetings. Select community members, providers, and AAC members joined Community Advisory Groups serving as co-evaluators of the program. Adapting global evidence-based approaches implemented by CARE, Save the Children, and UNICEF for humanitarian contexts, the AMAL Initiative engaged multiple stakeholders in an iterative participatory development process.

To determine the efficacy of the AMAL Initiative, CARE conducted an evaluation of this pilot implementation in northwest Syria to assess how the AMAL curriculum and multi-component approach contributed to changes in participants' knowledge of and attitudes toward reproductive healthcare, shifts in social norms affecting adolescent girls, and meaningful engagement of adolescents in programming. This paper describes the results of this evaluation.

## Materials and Methods

### Intervention

**Figure 1:**
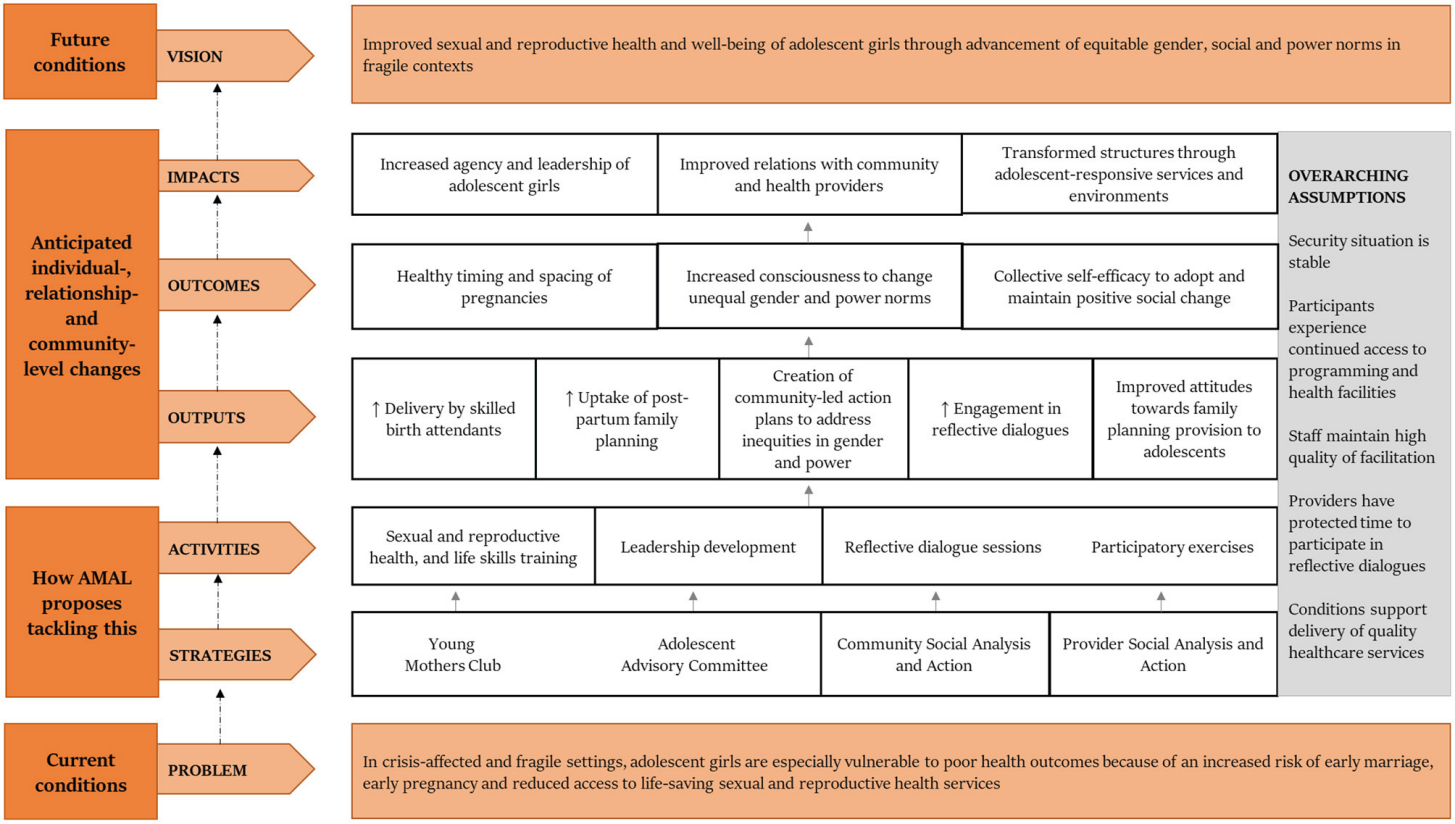
The AMAL initiative's theory of change.

The AMAL Initiative was carried out from January to December 2020 in the Azmarin and Abin villages in northwest Syria. A complete set of weekly sessions for each component was referred to as a cycle. Cycles comprised eight sessions for Young Mothers' Club (YMC), seven sessions for community, and four sessions for health providers. YMC, community, and health provider cycles occurred concurrently. Each YMC and community cycle was facilitated by a trained health provider. Each health provider cycle was facilitated by trained AMAL staff. Each YMC group was made up of six to eight participants and met in a verified safe space. Community groups comprised 15–20 participants. Health providers groups typically had 6–10 participants and met on-site at clinics. The YMC curriculum included education and exercises on contraception, birth planning, safe pregnancy, postpartum care, newborn care, violence prevention, effective communication, interpersonal relationships, and critical thinking. The community curriculum included dialogues on puberty, early marriage, power relations, gender-based violence, family planning, and household decision-making. The health provider curriculum included dialogues on right-based family planning, adolescent-friendly services, communication skills, and counseling. The full AMAL Initiative curriculum is publicly available on CARE's website. The overarching assumptions and anticipated outcomes of the AMAL Initiative are described in the theory of change (Figure 1).

### Participant Recruitment

Having worked in Azmarin and Abin for several years, Syria Relief and Development (SRD) leveraged its existing relationships in the region to promote the AMAL Initiative and recruit participants. The Young Mothers' Club was designed for first-time mothers and pregnant girls between the ages of 10 and 18 years. This group was intentionally defined due to their unique sexual and reproductive health needs and risks in emergency settings. Following the first cycle of YMC, word of the AMAL Initiative spread within the community and demand for participation increased. SRD's pilot implementation ultimately included girls up to 25 years because individuals outside of the intended target group continually expressed substantial interest in participation, specifically women above 19 years of age. YMC graduates with self-expressed interest to contribute toward the evaluation and improvement of the AMAL Initiative were recruited to the Adolescent Advisory Committee. For the community component of the AMAL Initiative, SRD conducted participant outreach within its previously established community protection networks. For the provider component, SRD recruited health providers from the sexual and reproductive health clinics and outreach sites that it supports.

### Study Design

This evaluation employed mixed methods to determine the effectiveness of the AMAL Initiative. The primary data collection tools were session evaluations, baseline and end line surveys, and focus group discussions. Session evaluations were conducted at the end of each session in a cycle in the form of group reflection to collect qualitative feedback on what went well and poorly and identify opportunities for improvement. All participants completed a quantitative baseline survey at the start of each cycle and an end line survey at the end of it. Serving as a pre-post measurement of outcomes, these surveys explored changes in attitudes and behaviors related to gender and power norms. As listed in Table 1, specific sets of survey questions correlated to overarching domains of change. For example, to determine changes in girls' self-esteem, girls reflected on five survey statements such as “I am a person of worth” and “I feel happy about who I am” using a five-point Likert scale. The end line survey included open-ended qualitative questions to collect participants' perceptions of the impact of AMAL on their well-being, relationships, and lives in general. At the end of the year-long AMAL Initiative, CARE carried out focus group discussions with session facilitators and SRD staff to collect their perspectives on potential areas for program improvement. To maintain COVID-19 safety protocols, focus group discussions were conducted using the Zoom meeting application. All participants engaged in informed verbal consent. No personal identifiers such as names and titles were recorded. Together these multiple mechanisms generated a body of robust qualitative and quantitative program evaluation data.

**Table 1:** Survey measures corresponding to each domain of change for each target population.

**Population**	**Domain of change**	**Corresponding measures *(Participants responded to each statement on a 5-point Likert scale at baseline and end line)***
Girls	Self-esteem	On the whole, I feel happy about who I am
		I have much to be proud of
		I am able to do things as well as most other people
		I am a person of worth, on equal plane with others
		I have respect for myself
	AAC girls' leadership skills	I am comfortable voicing my opinions to others
		I feel able to represent the voices/needs of others
		I identify as a leader among my peers
	Ability to communicate with family members	I can share my thoughts and feelings with my family
		I can start a discussion on family planning with my husband
		I can tell my husband if I wanted to use family planning
		My husband knows that I am currently using a method of family planning
	Confidence in seeking healthcare	I can advocate for my health needs
		I can discuss any problems or questions with the provider without feeling embarrassed
		I can seek health services at the health facility whenever I want
		I can seek and access health services even without permission from family
		For my next delivery (childbirth), I will have a health worker present or go to the health facility
	Relationships with family members	I feel valued within and by my family
		I am able to make some decisions in my household
		I can negotiate timing of sex or pregnancy with my husband
		My husband and I both have responsibilities in taking care of the children
Community members	Ability to recognize unequal gender norms	A man should have the final say about decisions in his home
		A wife should always obey her husband
		It is the mother's responsibility to take care of the children
		I can recognize unequal gender norms for boys and girls
		I believe that the lives of women and girls in my community could be improved
	Interest in changing unequal gender norms	I am interested in making changes in my family for gender equality
		I want to influence positive change within my community
		I am interested in making my community more equal for girls
		I am actively working to make my community more equal for girls and women
	Support for girls' equal access to services	A girl should be free to go outside the house when she wants
		A girl should be allowed to choose her husband
		A girl needs her husband's or mother-in-law's permission to go to the clinic
		A health provider should inform a man if his wife seeks any services
		Women should not seek work outside the home
		Women who work outside of the home are not good mothers or wives
	Support for girls' use of family planning	Young people should not have sex before marriage
		After marriage, girls should have children as soon as possible
		Family planning should only be available to married women
		A woman should not use family planning until she has had at least three children
		A woman does not have the right to use family planning just because she wants to
		If a woman wants to avoid being pregnant, she needs her husband's permission to use contraception
		If a woman wants to avoid being pregnant, it is her responsibility alone
		Only when a woman has a child does she become a real woman
	Support for girls' education	All girls should learn how to read and write
		Once a girl is married, she should leave school
		Once a boy is married, he should leave school
	Support for early marriage	Girls should marry as soon as possible to protect their chastity
		The ideal age of marriage for a girl is __ *(participants filled in)*
		Girls should be married before the age of 18
Healthcare providers	Beliefs supporting girls' choice to use family planning	Providing contraception to unmarried adolescents encourages adultery and promiscuity
		Girls should have their first child before the age of 19
		Young people should all receive contraceptive counseling, whether they are married or not
		A girl should be able to use family planning if she wants to
		Young women without children should be allowed to use any family planning product
	Influence on girls' ability to exercise reproductive rights	Adolescents are still young and immature so sometimes I need to make decisions for them
		It is part of my job to make sure myself and my colleagues provide appropriate reproductive health services to adolescents.
		Providers have a professional responsibility to listen and try to understand the needs of adolescent clients with respect
		Providers should talk about family planning with all adolescent clients, even if they are seeing the provider for a different health service.
		Talking about our values, beliefs, and professional responsibilities can help our facility provide better services to adolescent clients.
	Comfort delivering family planning services to adolescents	I enjoy working with adolescent clients
		I am equally comfortable providing family planning to an unmarried boy as to an unmarried girl
		I would give an IUD to an adolescent girl who had no children if she wished
		I would provide a hormonal method to a young client even if I knew it might damage my reputation in the community
		At times it can be embarrassing for me to discuss sex or family planning with younger patients
		If a few young girls have a negative experience with a method, I avoid recommending that method to other young clients
		There are reproductive health services that I will never give to adolescent clients
	Commitment to confidentiality of health services	Providers should keep information shared by adolescent clients confidential
		A provider must require the authorization of a girl's husband before providing her with family planning
		If a young girl asks for family planning, it is my responsibility to inform her husband/family that she is asking for that

### Data Analysis

For the baseline and end line surveys, all quantitative questions employed either a binary or a five-point Likert scale. Each ranking on the Likert scale was assigned a score between one and five, with one indicating strong disagreement and five indicating strong agreement. Higher scores represented greater adherence in attitudes/behaviors to the given individual or social norm. To assess changes in outcomes between baseline and end line, the analysis consolidated the scores across the set of questions relevant to each domain of change or topic area of interest. For example, to determine the impacts of participation in AMAL on girls' self-esteem, average scores for all participants were consolidated across the following five statements: I feel happy about who I am; I have much to be proud of; I am able to do things as well as most other people; I am a person of worth on equal plane with others; I have respect for myself. The consolidated baseline and end line scores for each domain are depicted in the figures in the Results below and converted to percentages in the text for ease of description. For the session evaluations and focus group discussions, transcripts were repeatedly and carefully reviewed to inductively develop a coding structure. The first round of review explored participant's general perceptions of the AMAL Initiative model and potential areas for improvement. Based on that reading and the evaluation objectives, an initial set of codes was developed and then honed through further data review. Subsequent line-by-line coding of transcripts focused on descriptions of impacts on girls' agency, relations between community and providers, and community structures and environments. All coding was done in MAXQDA Standard, a software tool designed for coding qualitative data. Following an iterative coding and memo process, two of the authors jointly developed a codebook. Once the full dataset was coded, data were further sorted by broader themes that had emerged.

## Results

Over 1 year of implementation in northwest Syria, 104 first-time mothers and pregnant girls, 219 family and community members, and 120 health providers participated in the AMAL Initiative. Of the 104 Young Mothers' Club participants, 33 girls received additional leadership training and served as leaders and program contributors via Adolescent Advisory Committees. The average age of YMC participants in this implementation was 17 years. Of all the YMC participants, 37% were above the age of 19, 60% were married with no children, 22% had one child and 18% had two or more children. Program evaluation results and participant reflections are organized below by the three program populations: adolescent girls, community members, and health providers.

### Outcomes Among Adolescent Girls

**Figure 2:**
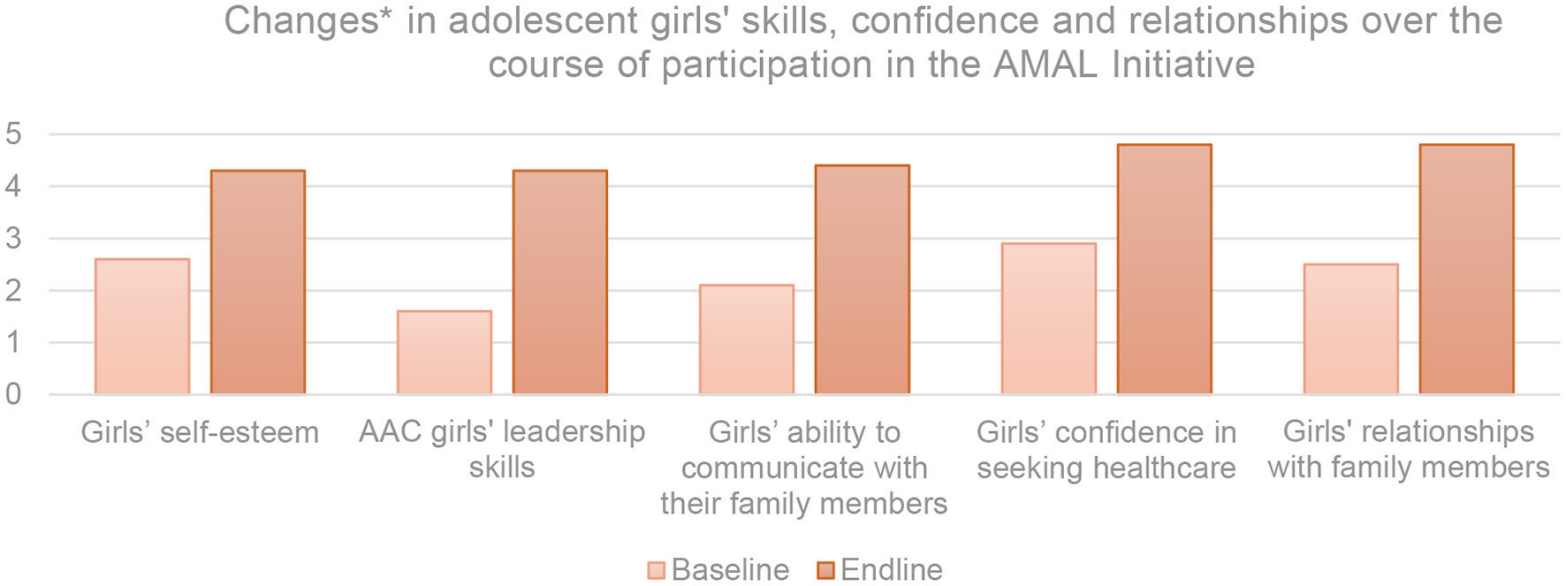
Consolidated changes between baseline and end line in AMAL adolescent participants' self-report of skills, confidence, and relationships on a five-point Likert Scale. *Measured through a consolidated analysis of norms statements using five-point Likert Scale wherein 1, strongly disagree; 2, disagree; 3, neutral; 4, agree; and 5, strongly agree.

The AMAL Initiative sought to facilitate increased agency of adolescent girls by including them in its design and evaluation, and delivering sessions intended to improve their skills, knowledge, and self-esteem. The changes that YMC participants reported across these areas between baseline and end line are illustrated in Figure 2.

Adolescent girls reported a 39% increase in self-esteem. Participants reported increases in feelings of self-worth, self-respect, and personal happiness. One YMC participant remarked that she saw herself “in a positive way now. It's harder for others to change that, and I can persuade my family to see that value too”. YMC participants who underwent additional curriculum and training in Adolescent Advisory Committees (AACs) reported a 128% increase in their leadership skills. Having had platforms and opportunities to contribute to the program and liaise with adults in their communities, AAC participants' self-assessments were particularly strong. One AAC participant said “I used to be afraid of my situation, but now I think I'm the strongest among my family. Before it felt like I didn't have a voice. I'm changed and can do anything”. Facilitators discussed how these changes to girls' self-esteem were noticeable to those around them and may have contributed to the increased community demand for opportunities for participation in the AMAL Initiative. Connected to girls' self-esteem was their ability to effectively communicate their thoughts and needs, and confidence to access services. Girls reported an 83% increase in their ability to communicate with their family members, and a 59% increase in their confidence in seeking healthcare. Commenting on the value of the knowledge that the AMAL curriculum had provided, one YMC participant said: “I understand my body more and I will insist on getting healthcare when I need it”. While there was an overall increase in girls' confidence to seek healthcare after participation in the program, scenarios involving lack of spousal permission had negative shifts between baseline and end line. Girls reported being unable to or unwilling to visit a health facility without the permission of their husbands at higher rates after participation in YMC. Despite this, adolescent girls reported a 60% improvement in their perceived quality of their familial relationships. Reflecting on how this has affected her household life, one AAC participant shared: “I've learned how to talk to my husband and family about what I want, and they agree that I should be included in making some decisions”.

### Outcomes Among Community Members

**Figure 3:**
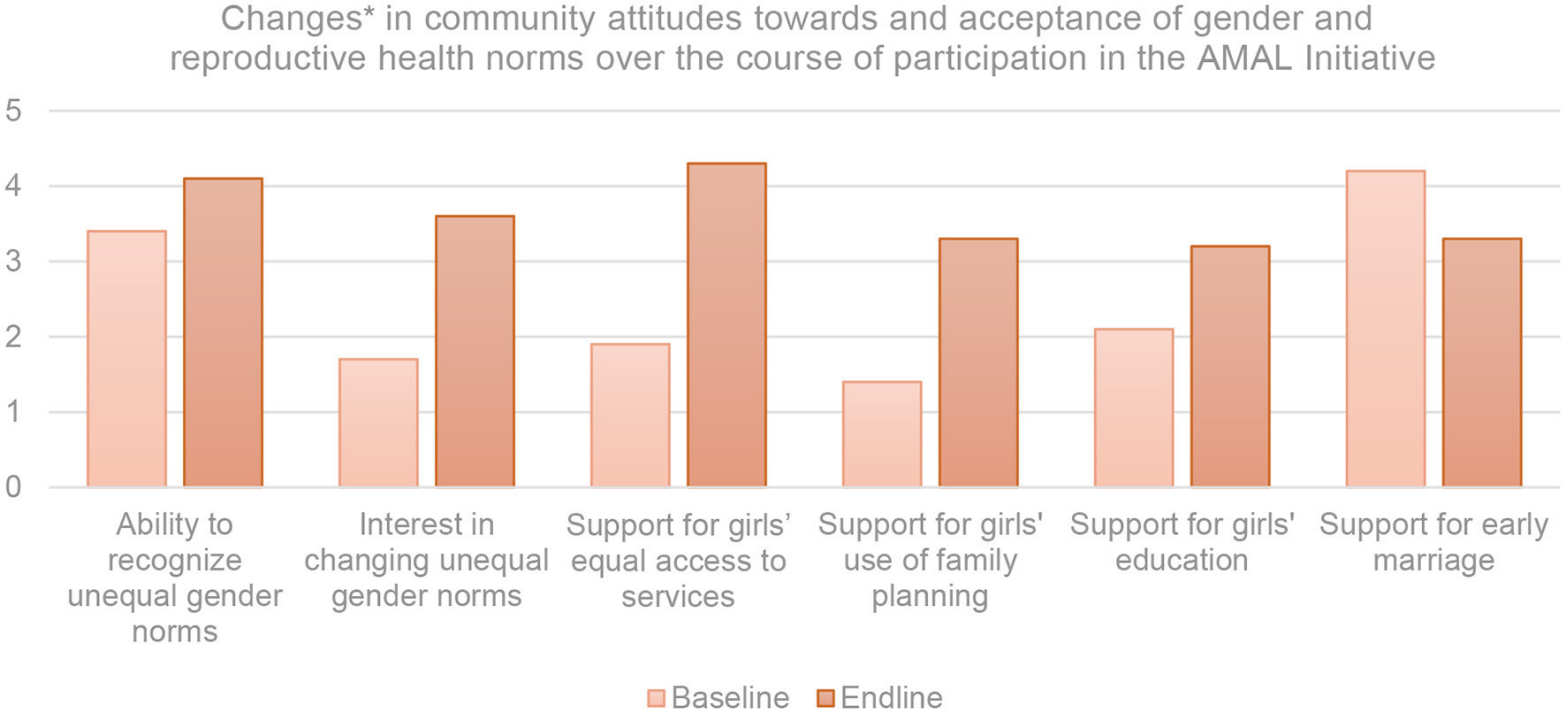
Consolidated changes between baseline and end line in AMAL community participants' self-report of attitudes toward and norms related to gender and reproductive health norms on a five-point Likert Scale. *Measured through a consolidated analysis of norms statements using five-point Likert Scale wherein 1, strongly disagree; 2, disagree; 3, neutral; 4, agree; and 5, strongly agree.

Recognizing the influential role that adolescents' familial and community networks play in their upliftment and well-being, the AMAL Initiative engaged girls' husbands, mothers-in-law, religious leaders, and influential community members in programming through the community component. The changes observed in community attitudes toward gender and reproductive health norms between baseline and end line are illustrated in Figure 3.

Indicative of adolescent girls' cited shifts in household dynamics, evaluation results found a 14% increase in community members' ability to recognize unequal gender norms and a 41% increase in community interest in changing unequal gender norms. Community members claimed this was facilitated by the active role that AMAL encouraged them to take in the support of adolescents. Participants repeatedly discussed experiencing feelings of responsibility toward and ownership over actions to encourage more equitable environments within their communities. This increased stake in and desire for equality appeared to carry over into other areas. Community support for girls' equal access to services increased by 35%. This included services related to healthcare, social support, and education. Since girls' ability and willingness to seek out healthcare can be affected by those around them, the evaluation explored community views toward family planning. Increasing overall by 27%, community support for girls' use of family planning was positively correlated with the number of children a married girl had previously birthed. For girls with three or more children, all participants surveyed strongly supported the use of family planning but for girls with fewer than three children, the intensity and amount of community acceptance of family planning decreased. Support for use of family planning was lowest in scenarios that described girls engaging in contraception for no reason other than because they had the choice to do so. Of all services, girls' equal access to education garnered the lowest level of community support, especially when discussing situations of a girl's education post marriage. Community support for girls' education overall increased by 39% but decreased to 12% for married girls. Evaluation findings suggest a lower individual and community tolerance for child and early marriage, with community support for early marriage decreasing by 38%. Community participants indicated a shift in attitudes toward greater recognition of the issues posed by early marriage for girls, and a trend toward decreased acceptance of the practice.

Participants commented on how the design of the AMAL Initiative enabled its success. They noted that having men and male religious leaders participate was important for changing social norms, especially related to marriage. Commenting on community attitudes toward early marriage, an AMAL facilitator said: “We noticed a huge change in the mentality of men after attending community sessions. An Imam (religious leader) said that the highest divorce rate is among adolescents, and that influential people in the community should be doing something about it by raising awareness about the wrongness of child marriage”. This observation showcases the role that men and religious leaders play in upholding or altering the status quo in this community. Facilitators believed that while previous efforts related to sexual and reproductive health programming with adolescents were received poorly, AMAL's multi-component approach and participatory nature resulted in community buy-in. The AMAL Initiative's intentional inclusion of individuals that affect the lives of married girls, such as their mothers/mothers-in-law, husbands, and health providers was crucial to its acceptance by the greater community.

### Outcomes Among Health Providers

**Figure 4:**
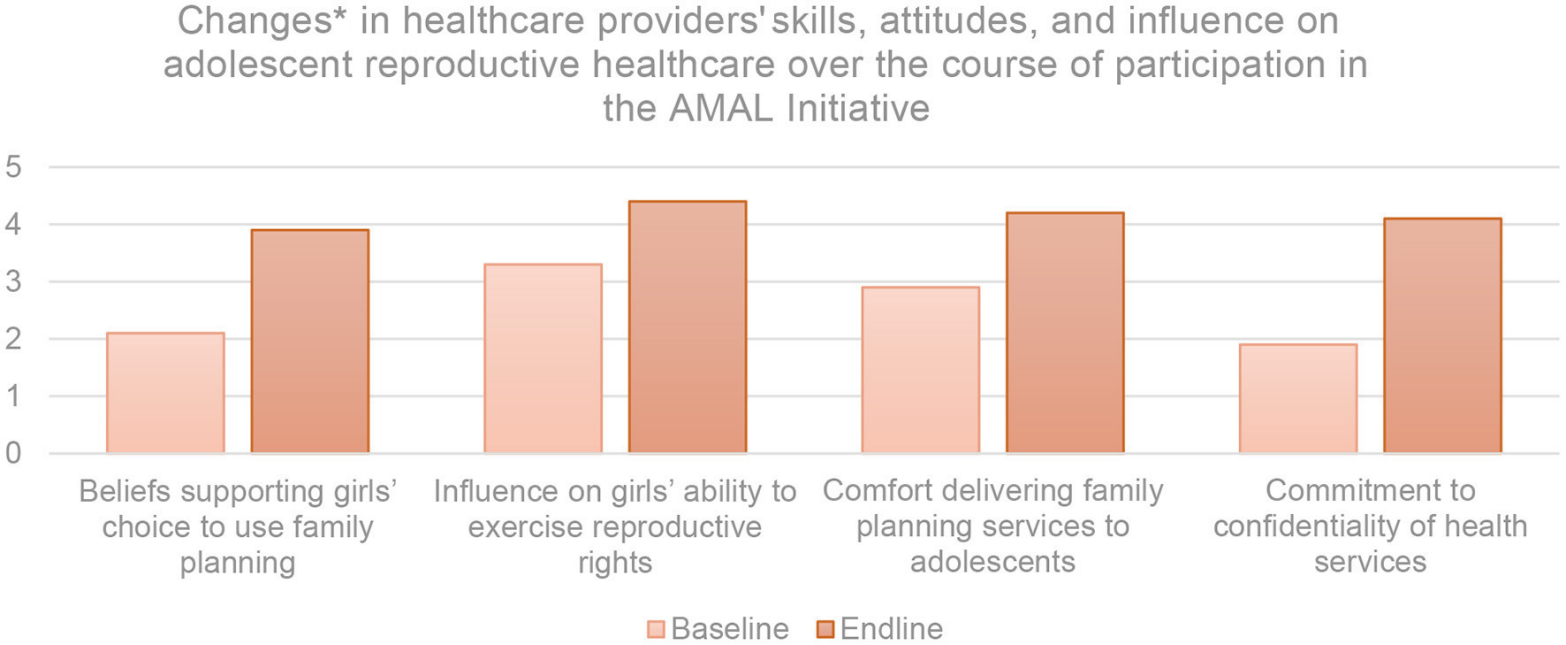
Consolidated changes between baseline and end line in AMAL healthcare provider participants' self-report of their skills in, attitudes toward, and influence on adolescent reproductive healthcare on a five-point Likert Scale. *Measured through a consolidated analysis of norms statements using five-point Likert Scale wherein 1, strongly disagree; 2, disagree; 3, neutral; 4, agree; and 5, strongly agree.

Adolescent-friendly transformation and responsiveness of health services is greatly determined by the attitudes of the individuals providing those services. The AMAL Initiative evaluation included an assessment of changes in providers' approaches to adolescent sexual and reproductive healthcare between baseline and end line. These are illustrated in Figure 4.

Beliefs among providers that girls have the right to choose to use family planning increased overall by 44%. Providers' belief and understanding that they have influence on girls' ability to exercise their reproductive rights increased by 33% and their comfort delivering family planning services to adolescents increased by 31%. This was bolstered by qualitative evaluation findings with most providers claiming they had an improved cognizance of how to speak to, respect the wants of, and deliver services to adolescent girls. A health provider shared that they “understand the health needs of adolescents now and can talk to girls in a better way than before”. When asked about the significance of their participation in AMAL, one provider remarked: “[AMAL sessions] have really helped my practice and how I can deal with adolescents' reproductive care, especially for girls. I got a lot of new information and can see how the power and gender dynamics between spouses affects their own health, and how I can affect that”. In accordance with this new understanding and recognition of rights-based care, providers' commitment to ensuring confidentiality of health services increased by 46% overall. However, evaluation results suggested some level of dissonance in providers' reports of belief in choice and confidentiality. Despite all providers strongly acknowledging a commitment to confidentiality, some providers responded that in a scenario where a girl wanted family planning without her husband's presence or permission, they would tell the girl's husband about her visit to the clinic. When interrogated about how these ideas are contradictory, provider's explanations were varied. Some said that the AMAL Initiative had taught them that family planning needed to be a joint responsibility, and others cited gender norms of husbands remaining the decision-makers for all things affecting a household. When reflecting on sessions with providers, facilitators shared having to tackle some misconceptions on the use of long-acting reversible contraceptives at the start of program cycles. For example, there was a strong belief among providers that intrauterine devices would cause infertility and so a woman should not use them until she had birthed at least three children. While evaluation results at end line showed improvements in provider knowledge about contraceptive methods, facilitators suggested expanding the provider-specific curriculum to include more information about contraception myths and misconceptions.

## Discussion

The AMAL Initiative's vision was for improved sexual and reproductive health and well-being of adolescent girls through advancement of inequitable gender, power, and social norms. In designing the program and road mapping potential pathways to achieve this vision, CARE hoped to achieve impact across the individual, relationship, and community level. Per the AMAL Initiative theory of change, this translated to three impact areas: increased agency and leadership of adolescent girls, improved relations with community and health providers, and transformed structures through adolescent-responsive services and environments. Generating and sustaining improvements to adolescents' lives and well-being requires transforming structures, services, and environments to be responsive to adolescents' needs. By addressing community consciousness around gender, power, and social norms, the AMAL Initiative sought to create an adolescent-enabling context in its implementation areas. Increased support for family planning, decreased acceptance of early marriage, improved provision of adolescent reproductive healthcare, and greater involvement of adolescents in their communities suggest a more supportive and enabling environment for adolescent girls and an advancement in equitable norms. Evaluation results show that participatory adolescent-centered sexual and reproductive health programming to improve the self-efficacy of vulnerable adolescents to access healthcare and improve well-being is feasible in crisis settings.

Multiple studies have recognized the need for comprehensive approaches and packaging of adolescent sexual and reproductive health in crisis settings. Engel et al. highlight the importance of integrated promotive, preventive, and curative interventions for adolescent health (17). Particularly for interventions targeting norms around child, early and forced marriage, layering response, promotion, and prevention can be difficult (18). The AMAL Initiative suggests a possible structure for this–The curriculum was developed in response to increasing rates of adolescent marriage and pregnancy, and repeatedly addressed these topics with participants. While community and provider dialogues promoted shifts in gender norms, elevating girls' voices and assigning community stakeholders responsibility generated a social environment that could facilitate long-term prevention. Because of the participatory development of the AMAL Initiative, its design organically amalgamated evidence and promotive and preventive approaches from several other contexts. The AMAL Initiative's YMC curriculum was adapted from Save the Children's My First Baby, CARE's TESFA, and UNICEF Iraq's Adolescent Girl program (19). The community and provider change components utilize CARE's Social Analysis and Action and IMAGINE programming (20). Developed in line with the Whole of Syria adolescent strategy, and in collaboration with northwest Syria's gender-based violence and sexual reproductive health sub-clusters, the AMAL Initiative's intervention package is deeply integrated with and overlaid onto global approaches. Operating at multiple levels of the socioecological framework is a promising approach to creating enabling environments for adolescent sexual and reproductive health (21). By increasing girls' agency, improving relations with community and providers, and transforming structures, services, and environments, the AMAL initiative had outcomes at individual, relationship, and community levels. Per Svanemyr et al.'s proposed ecological model for changing environments, the AMAL Initiative influenced underlying determinants of behavior at all levels except societal as the program did not include any legal or policy advocacy or governance.

Global guidance for ethical humanitarian response notes the importance of including participatory approaches in programming for adolescents. To ensure as responsible and responsive an intervention as possible, intentional thought and consideration was given to global humanitarian principles during the design of the AMAL Initiative. It applies three of the Core Humanitarian Standards relating to participation and accountability: response is based on communication, participation, and feedback; complaints are welcome and addressed; and actors continuously learn and improve (1). The end-of-session evaluations and monthly community feedback meetings facilitated an ongoing assessment and continuous adaptation and improvement of the program to meet participants' needs. The Compact for Young People in Humanitarian Action outlines the following actions for humanitarian organizations: service delivery; participation; capacity and local action; resources; data and knowledge (22). The AMAL Initiative addresses each of these actions in at least some capacity. It ensures service delivery through the AMAL curriculum sessions on improve knowledge of family planning, participation through the AACs, capacity and local action through additional leadership training for girls and action planning for community members, resources through health provider training, and data and knowledge through continuous evaluation and a focus on learning. The AMAL Initiative also addresses some of the research gaps identified in the Interagency Working Group's adolescent sexual and reproductive health in humanitarian settings toolkit (23, 24). Specifically, it addresses the unique needs of married adolescents, describes a platform through which to engage youth as first responders or contributors, and meaningfully engages adolescents in the implementation and evaluation phases of the program cycle.

### Recommendations for Adolescent-Responsive Reproductive Health Programming

The AMAL Initiative is intended for humanitarian practitioners working in crisis-affected and fragile settings with the goal of supporting married adolescents to improve their sexual and reproductive health and overall well-being. This evaluation has demonstrated encouraging results across most domains for all components of the AMAL Initiative. Informed by this pilot iteration of the AMAL Initiative, below are some overarching recommendations for designing, implementing, and evaluating adolescent-responsive reproductive health programming in humanitarian settings:

- While adolescents holistically have needs that necessitate consideration during program design, various subgroups with unique vulnerabilities exist within this population. It is important for program implementers to identify the subgroup for which they are intending to generate change and cater their programming for the subgroup's characteristics. The AMAL Initiative targeted pregnant girls and first-time mothers, designing its curriculum to primarily address topics related to pregnancy, antenatal care, birth, postnatal care while packaging in discussions of choice, agency, and self-esteem.
- Creating avenues for adolescent involvement in program design and/or delivery is critical to honoring the voices of young people in crisis-affected contexts and ensuring truly responsive programming. Due to the constraints that humanitarian programs typically operate within, it can be challenging to design activities that intentionally, actively, and continually consider the opinions of young people. However, participatory approaches recognizing not only the unique needs of adolescents, but also their capacity to influence change for themselves are especially effective for generating positive change (21). The AMAL Initiative has demonstrated some success in this area by using participant feedback to improve subsequent program cycles and providing a platform for girls' voices and leadership through the AACs.
- Generating positive change in attitudes, behaviors and norms is best facilitated by a multi-component approach that engages not just at-risk adolescents but their familial and societal communities in programming. As evidenced by participant feedback and facilitator reflections, much of the AMAL Initiative's success in securing buy-in and shifting community consciousness around gender and social norms is attributable to its direct engagement of individuals within adolescent girls' networks. Levying a sense of personal responsibility for change on community members through dialogues and action planning contributed to the creation of enabling environments for adolescent healthcare and well-being.
- Building in mechanisms for ongoing evaluation, such as rapid feedback assessments, creates a recurring opportunity to engage in continuous quality improvement and gather stakeholder opinions, including adolescents, on the relevance of a program. In humanitarian situations, the nature of a crisis can shift quickly based on several factors. Regular monitoring and evaluation provide an analysis of the situational context and assist with adaptation. Even in a protracted crisis like that of Syria, the sudden onset of the COVID-19 pandemic necessitated shifts in programming that the AMAL Initiative was able to accomplish in part due to its weekly evaluation schedule.

### Areas for Improvement and Future Directions

Young people, including adolescents, are especially vulnerable in times and areas of conflict and crisis, but are not often prioritized in intervention design or effectively reached during implementation. Even when programs target adolescents for inclusion, they typically engage older adolescents rather than younger ones or a full range of participants between 10 and 18 years of age (25, 26). While unintended, this turned out to be the case for this pilot implementation of the AMAL Initiative in Azmarin and Abid. Originally designed for pregnant girls and first-time mothers aged 10 to 18 years, it was challenging to recruit younger girls into the program due to significant community pressure and demand for participation for older adolescent and young women above the age of 19 years. Implementers at Syria Relief and Development decided to adapt the program inclusion criteria for YMC to include participants under 25 years of age. There is a possibility that recruitment of younger adolescents was limited because the number of girls aged 10 to 14 years in Azmarin and Abid that met the program criteria of being pregnant or mothers was minimal, but CARE does not have any strong evidence to reach that conclusion. Adolescents ages 10 to 18 years undoubtedly have unique needs that are extremely worthy of consideration in program intervention design, but the demand that the AMAL Initiative experienced from individuals above 18 years highlights how in contexts such as Syria, sexual and reproductive health-related gender and social norms continue to affect older, married girls and points to the need for integration of varying types of age-appropriate programming for girls and young women.

Actualizing adolescents' potential to realize their individual capacity and collective efficacy is necessary for adolescents to be able to influence and improve their lives. AMAL's adolescent participants experienced improvements to their self-esteem and their perceived ability and confidence to enact change for themselves across many areas of their lives. Seeing the value in their opinions and selves motivated the participating girls to seek out better communication, relationships, and access to services. However, a surprising change in participant attitudes observed between baseline and end line was that more girls reported being unable to or unwilling to visit a health facility without the permission of their husbands. While it is possible that their response to this question was influenced by a feeling that their husbands should be in attendance for any visit to a health facility because family planning is a joint responsibility, it is difficult to discern that with our current data. In addition to empowering girls to actualize their self-efficacy for change, the AMAL Initiative intended to improve community members' and health providers' attitudes related to adolescent reproductive healthcare. While it appears to have achieved this overall, some areas improved more significantly than others. For example, attitudes toward unmarried women and women with less than three children having access to family planning significantly improved based between baseline and end line. Attitudes toward a woman having the right to choose family planning simply because she wants to, however, had minimal improvement. Health providers too saw spousal permission as an exemption to their otherwise strong increases in beliefs about girls' reproductive choice and confidentiality of care. These findings collectively indicate that the AMAL Initiative may have been less effective in shifting norms relating to gendered roles within a family or household, particularly around reproductive decision-making. Future programming for young adolescents intending to protect their reproductive rights or improve equitability of reproductive decision-making might consider other solutions to this as an area for further exploration.

### Limitations

As the AMAL Initiative sought to increase community consciousness of inequitable norms and collective self-efficacy to adopt positive social change, this evaluation collected information on participants' self-reported changes in attitudes and behaviors related to particular gender, social, and power norms. It is however limited in its ability to track how these changes in perception of norms translated into health or social outcomes within the communities participating in the AMAL Initiative. Collecting data at local health facilities on the number of family planning visits, uptake of family planning methods, birth timing and spacing, and antenatal and postnatal visits could facilitate a more robust picture of the impact of this intervention on individual and population sexual and reproductive health outcomes. Future iterations of the AMAL Initiative will seek to include facility-level sexual and reproductive health data. Similar outcome data on the number of early and forced marriages, girls' education attendance, and distributions of household labor is required to track sustained changes in norms-related practices.

Since the AMAL Initiative was primarily focused on affecting the sexual and reproductive health of pregnant girls and first-time mothers, the curriculum did not include sessions on girls' inclusion in household decision-making or education. While these originally seemed extraneous to the program's intention, the observed results around the conflict between the AMAL Initiative's promotion of ideas of joint responsibility of family planning, and existing social norms of husbands being sole decision makers for households suggest that the inclusion of a session or curriculum content on shared decision-making would be beneficial.

## Conclusion

With the face of fragile contexts becoming increasingly young, the AMAL Initiative seeks to inform the global evidence base and dialogue around nexus approaches to adolescent-responsive sexual and reproductive health programming. Leveraging the lessons from this pilot implementation in Azmarin and Abid, the AMAL Initiative is being scaled up in Syria by eight local organizations and in northern Nigeria by CARE. Early results from Nigeria show significant positive changes in girls' self-esteem, norms relating to adolescent girls' use of family planning, and support for practice of healthy spacing of pregnancies, but limited impact on early marriage practices. Over these subsequent implementations, CARE hopes to continue to identify what is and isn't effective toward meeting the unique needs of vulnerable sub-groups of adolescents in crisis settings. Such an ongoing investment in learning is necessary for organizations like CARE and others as well as humanitarian practitioners to ensure relevant, fruitful, and empowering programming for adolescent sexual and reproductive health.

## Data Availability Statement

The raw data supporting the conclusions of this article will be made available by the authors, without undue reservation.

## Ethics Statement

Ethical review and approval was not required for the study on human participants in accordance with the local legislation and institutional requirements. Written informed consent from the participants' legal guardian/next of kin was not required to participate in this study in accordance with the national legislation and the institutional requirements.

## Author Contributions

PC oversaw data collection procedures and contributed to the conceptualization, preparation and editing of the manuscript, and analyzed the data and identified recommendations. AK supported with detailed review and contribution. FM and IA provided broader review. IA coordinated the data collection and the local research group and conducted some of the focus groups. AK and PC co-supervised the study and AK and FM provided thought leadership on study directions, effectiveness, and relevance. All authors have made significant contributions to the AMAL Initiative and this manuscript, accept responsibility for its content, and read and approved the final manuscript.

## Funding

Since its inception, the AMAL Initiative has received funding from the United Nations Population Fund and USAID's Bureau of Humanitarian Assistance.

## Conflict of Interest

The authors declare that the research was conducted in the absence of any commercial or financial relationships that could be construed as a potential conflict of interest.

## Publisher's Note

All claims expressed in this article are solely those of the authors and do not necessarily represent those of their affiliated organizations, or those of the publisher, the editors and the reviewers. Any product that may be evaluated in this article, or claim that may be made by its manufacturer, is not guaranteed or endorsed by the publisher.
